# Biological sex and disease architecture in systemic sclerosis: a multilayer translational framework for precision medicine

**DOI:** 10.3389/fimmu.2026.1978654

**Published:** 2026-10-05

**Authors:** Yi’an Tian, Zhaofang Tian

**Affiliations:** 1 Department of Rheumatology and Immunology, The Affiliated Huai’an No.1 People’s Hospital of Nanjing Medical University, Huai’an, China; 2 Department of Neonatology, The Affiliated Huai’an No.1 People’s Hospital of Nanjing Medical University, Huai’an, China

**Keywords:** autoimmunity, biological sex, disease architecture, multi-omics, precision medicine, sexual dimorphism, systemic sclerosis

## Abstract

Biological sex is routinely incorporated into systemic sclerosis (SSc) research as a demographic or stratification variable. Women are substantially more likely to develop SSc, whereas men experience a greater burden of severe organ involvement and adverse outcomes. Whether these observations represent parallel sex-associated differences or reflect differences in the relationships among disease components remains unresolved. Here, we propose the Systemic Sclerosis–Multilayer Architecture Framework (SSc-MAF) as a hypothesis-generating framework for addressing this distinction. We define a sex-dependent architectural difference not as an isolated difference in a molecule, cell population, pathway, or clinical phenotype, but as a reproducible sex-associated difference in relationships among disease components, including multicellular communication, fibrovascular coupling, spatial tissue organization, molecular co-activation, or longitudinal clinical–biological trajectories. SSc-MAF therefore distinguishes clinical disease trajectories from the biological architecture that may generate them and from the methodological strategies required for empirical validation. We critically evaluate current evidence across immune, fibrovascular, metabolic, and regulatory domains, while distinguishing direct sex-stratified SSc evidence from indirect mechanistic evidence and framework-level hypotheses. We further outline measurable predictions and conditions that would weaken or fail to support individual propositions of SSc-MAF. This framework is intended not to establish sex-dependent disease architecture as a proven feature of SSc, but to provide an operational strategy for testing whether biological sex contributes information beyond conventional component-level comparisons and additive covariate adjustment.

## 1 Introduction

Systemic sclerosis (SSc) is a heterogeneous autoimmune disease characterized by vasculopathy, immune dysregulation, and progressive fibrosis of the skin and internal organs (1, 2). These processes do not occur independently. Endothelial injury, innate and adaptive immune activation, fibroblast-state transitions, extracellular-matrix remodeling, and organ-specific tissue responses interact over time to generate markedly different clinical trajectories (1, 2).

Biological sex represents one of the most reproducible dimensions of SSc heterogeneity. Women constitute the substantial majority of patients with SSc (3, 4). In several observational cohorts, men have more frequently exhibited diffuse cutaneous disease and severe internal-organ involvement (5), while male sex has also been associated with reduced survival (5–7). Recent sex-stratified genetic evidence further indicates that susceptibility loci are not identical between women and men (8).

However, these observations create an unresolved conceptual problem. A difference in disease frequency, genetic susceptibility, gene expression, cell abundance, autoantibody prevalence, or clinical outcome demonstrates a sex association with that component; it does not establish that the relationships among components are themselves organized differently.

This distinction is increasingly important because contemporary SSc research is moving from isolated biomarkers toward multicellular and spatially resolved models of disease. Single-cell studies identify heterogeneous stromal and immune states (9–11), while spatial and multimodal studies reveal pathological vascular, fibroblast–macrophage, and other multicellular niches (12–16). These approaches make it possible to ask a question that conventional sex-stratified comparisons rarely address: does biological sex modify the coupling among disease processes rather than simply the magnitude of individual processes?

We therefore propose the Systemic Sclerosis–Multilayer Architecture Framework (SSc-MAF). In this framework, disease architecture refers to the reproducible relationships among molecular programs, cellular states, tissue compartments, and clinical trajectories. A sex-dependent architectural difference consequently requires more than a female–male difference in an individual variable. It requires evidence that a relationship itself—for example, immune–fibroblast communication, endothelial–stromal coupling, spatial neighborhood organization, cross-layer molecular covariance, or the association between a biological state and subsequent disease progression—differs according to sex.

SSc-MAF distinguishes three levels of evidence. Component-level evidence identifies sex-associated differences in individual clinical or biological features. Coupling-level evidence demonstrates that the relationship between two or more disease components differs by sex. Architecture-level evidence requires reproducible sex-dependent coupling across complementary datasets, biological scales, longitudinal observations, or experimental perturbations and, ideally, evidence that this relationship provides information beyond additive sex effects and established covariates.

The framework is deliberately falsifiable. If adequately powered and appropriately balanced studies demonstrate that multicellular interactions, spatial neighborhoods, cross-layer molecular relationships, and biological–clinical coupling are comparable between women and men after accounting for relevant covariates, the central architectural proposition would not be supported even if individual sex-associated differences remain detectable.

The purpose of this Review is therefore not to argue that distinct female and male SSc architectures have already been established. Rather, we critically examine the evidence that motivates this hypothesis, identify where evidence remains component-level or indirect, and define experimental and analytical strategies capable of testing whether biological sex contributes to the organization of SSc beyond conventional stratification. These distinctions form the conceptual structure of SSc-MAF (**Figure 1**).

**Figure 1:**
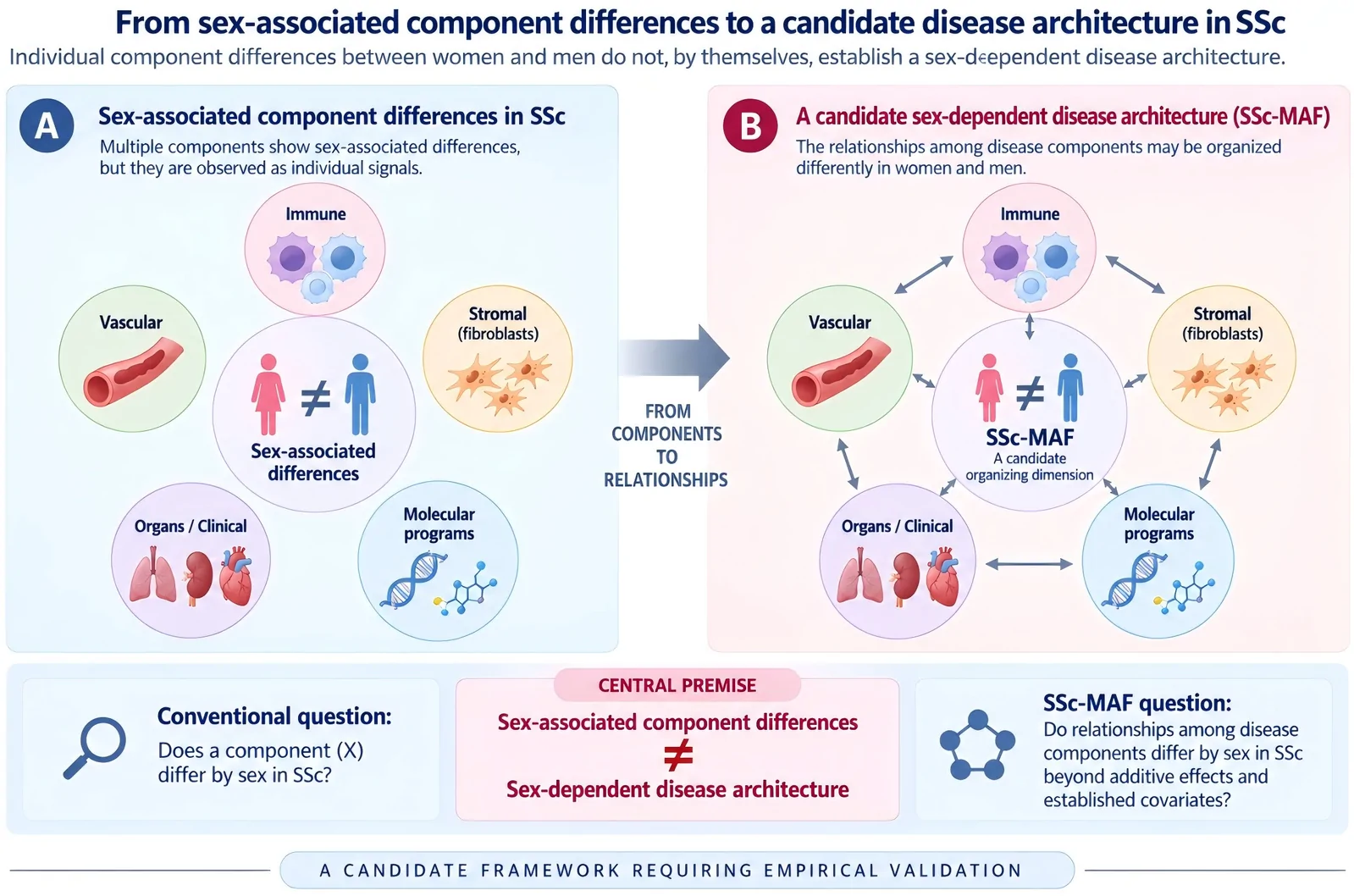
Conceptual framework of the Systemic Sclerosis–Multilayer Architecture Framework (SSc-MAF). **(A)** Sex-associated differences have been reported across multiple components of systemic sclerosis (SSc), including immune, vascular, stromal, molecular, and clinical domains. The magnitude and consistency of these differences vary across components, populations, and studies. Such component-level observations do not, by themselves, establish sex-dependent disease architecture. **(B)** SSc-MAF proposes biological sex as a candidate organizing dimension whose relevance should be tested at the level of relationships among disease components. Evidence for sex-dependent architecture would therefore require reproducible sex-associated differences in intercomponent relationships rather than the coexistence of multiple female–male differences. Arrows represent candidate relationships among components and do not imply established directionality or causality. Female and male symbols are used as conceptual representations of recorded biological/clinical sex and should not be interpreted as direct proxies for chromosomal, hormonal, reproductive, gender-related, or other contextual determinants. SSc-MAF is presented as a testable framework requiring empirical validation.

## 2 Literature identification and evidence classification

For this revised Review, literature was identified through targeted searches of PubMed/MEDLINE through 20 September 2026, supplemented by reference tracing from major SSc reviews, systematic reviews, cohort studies, and recent genetic, single-cell, spatial, and multi-omic studies. A representative PubMed search syntax was (“systemic sclerosis” OR scleroderma) AND (sex OR gender OR male OR female), with additional topic-specific searches combining the disease terms with survival, interstitial lung disease, pulmonary arterial hypertension, genetics, X chromosome, X-chromosome inactivation, TLR7, sex hormones, fibroblast, endothelium, single-cell analysis, spatial transcriptomics, spatial proteomics, epigenetics, metabolism, biomarkers, or treatment response. Particular emphasis was placed on human SSc studies, sex-stratified cohorts, primary mechanistic studies, and recent studies capable of resolving cellular, spatial, or cross-layer relationships. Reference lists of relevant reviews and primary studies were additionally examined to identify potentially informative studies not captured by the targeted searches.

Because SSc-MAF is a conceptual framework rather than a systematic review or meta-analysis, studies were not pooled quantitatively. Instead, evidence was classified according to its relationship to the framework: (A) direct sex-stratified SSc evidence; (B) SSc evidence without direct testing of sex dependence; (C) indirect mechanistic evidence from broader biological research; and (D) SSc-MAF hypotheses requiring prospective validation. This classification is used throughout the Review and in **Table 1** to prevent mechanistic plausibility from being presented as established sex-dependent SSc biology.

**Table 1:** Evidence map for sex-associated heterogeneity and candidate sex-dependent disease architecture in systemic sclerosis.

	Domain	Evidence cat.	SSc-MAF level	Key finding	Key limitation	Architectural implication	Ref.
Clinical phenotype and outcomes	Clinical phenotype / survival	A	1	Men more frequently had diffuse disease and ILD; male sex associated with mortality after adjustment.	Observational; residual confounding possible.	Component-level phenotype + trajectory evidence; does not establish sex-dependent architecture.	(5)
	Genetic susceptibility	A	1	8 sex-associated susceptibility loci identified (novel male- and female-associated signals).	Susceptibility genetics does not establish disease-state organization.	Strong component-level genetic evidence; architecture requires downstream coupling tests.	(8)
	SSc-ILD (trials)	A	1	Sex-associated pulmonary outcomes and biological profiles in SLS I/II.	Post-hoc; relatively few men.	Direct longitudinal phenotype evidence; motivates but does not prove coupling-level testing.	(17)
	SSc-ILD (tocilizumab)	A	1	Male sex associated with FVC decline in focuSSced/SMART.	Exploratory subgroup; no formal sex-by-treatment interaction test.	Exploratory treatment heterogeneity; requires formal sex-by-treatment interaction testing.	(18)
	SSc-ILD (EUSTAR)	A	1	Higher male mortality without uniformly faster FVC deterioration (n = 6,389).	Observational; cohort heterogeneity.	Demonstrates non-uniformity across outcomes; argues against a simple “male = worse architecture” narrative.	(19)
	Pulmonary phenotype	A	1	ILD more frequent/earlier in men; small-airway dysfunction more prominent in women.	Retrospective; single center.	Multidirectional component evidence; supports need for coupling-level analysis.	(20)
	SSc-ILD proteomics	A	1	Sex-associated protein differences but no significant sex difference in FVC-decline endpoints.	Exploratory; multiple testing.	Molecular difference ≠ divergent trajectory; key example of Level 1 without Level 2.	(21)
	SSc-PAH (PHAROS)	A	1	Male sex independently associated with mortality.	Limited sample (n = 131).	Organ-specific prognostic evidence; Level 1 only.	(22)
	SSc-PAH (cohort)	A	1	Male sex not independently predictive after adjustment.	Limited male subgroup.	Counter-evidence to a uniform male-risk model; Level 1, no architecture support.	(23)
Immune mechanisms	X-linked immune regulation	A	1	Altered TLR7/8 XCI in SSc pDCs with IFN-related heterogeneity.	Multicellular topology not tested.	Direct sex-linked component evidence; Level 2 requires network testing.	(26)
Tissue, spatial and multicellular coupling	Vascular niche (spatial proteomics)	B	1 (disease-level)	Pathological vascular neighborhoods identified in SSc skin.	Sex dependence untested.	Disease-level spatial coupling established; sex dependence remains a testable SSc-MAF hypothesis.	(12)
	Fibroblast–macrophage niche (spatial multi-omics + perturbation)	B	1 (disease-level)	Spatial dependency; CXCR4 inhibition attenuated fibrosis.	Small discovery cohort.	Spatial coupling with perturbational support; sex dependence remains untested.	(13)
	Fibroblast spatial states (ciSH)	B	1 (disease-level)	Spatially resolved fibroblast populations and pathogenic niches.	Sex dependence untested.	High-resolution spatial organization in SSc; sex dependence remains untested.	(16)
	Early vascular niche (multimodal)	B	1 (disease-level)	Perivascular macrophage–fibroblast signalling niche in early dcSSc.	Sex dependence untested.	Independent multicellular/spatial coupling; sex dependence remains untested.	(33)
	Immunofibrotic coupling (CCL19–CCR7)	B	1 (disease-level)	CCL19+ fibroblast–CCR7+ T-cell crosstalk with experimental validation.	Sex dependence untested.	Direct disease-level multicellular coupling; sex dependence remains untested.	(31)
	Endothelial–stromal coupling (ACKR1)	B	1 (disease-level)	ACKR1+ endothelium promotes leukocyte infiltration and fibroblast activation.	Sex dependence untested.	Mechanistically supported cross-compartment coupling; sex dependence remains untested.	(15)
	Regulatory / epigenetic programs	B	1 (disease-level)	DNA methylation and chromatin-accessibility abnormalities in fibroblasts/endothelial cells.	Direct sex dependence incompletely tested.	Candidate regulatory substrate; sex-dependent coupling remains untested.	(35, 36)
Hormonal, metabolic and other mechanisms	Sex hormones (systematic review)	B	1 (SSc component-level)	Heterogeneous vascular and fibrotic effects reported across human and experimental SSc literature.	Causality uncertain; direct sex-dependent coupling not established.	SSc-specific mechanistic evidence; sex-dependent architecture remains untested.	(38)
	TLR7 XCI escape (non-SSc)	C	—	Biallelic TLR7 expression in female immune cells.	Not SSc; indirect.	Indirect support for X-linked immune mechanisms; C-class evidence.	(27)
	Metabolism / macrophage polarization	C	—	Metabolic programs regulate macrophage polarization.	No direct SSc sex-dependent evidence.	Candidate regulatory layer; direct SSc sex-dependent coupling remains an evidence gap.	(37)
Framework hypothesis	Sex-dependent multicellular / spatial / cross-layer coupling	D	3 (hypothesis)	Systematic sex dependence remains unresolved.	Requires adequately powered sex-balanced studies.	Central SSc-MAF prediction; to be tested using the validation roadmap in **Figure 4**.	—
	Longitudinal biological–clinical coupling	D	3 (hypothesis)	Whether identical biological states predict different trajectories by sex remains unresolved.	Requires prospective longitudinal validation.	Central SSc-MAF prediction; requires formal sex-by-state and sex-by-time testing.	—

Evidence categories: A = direct sex-stratified evidence in SSc; B = SSc-specific evidence of disease-level coupling or mechanistic evidence without direct testing of sex dependence; C = indirect mechanistic evidence from broader biological research; D = framework-level hypothesis. SSc-MAF level: 1 = component-level evidence or established disease-level relationship without demonstrated sex dependence; 2 = sex-dependent coupling; 3 = sex-dependent architecture. A sex-associated difference in an individual component is not considered evidence of sex-dependent disease architecture unless relationships among disease components are shown to differ according to sex. No study identified in the literature reviewed here directly demonstrated sex-dependent coupling that met the prespecified criterion for Level 2; this absence represents a key testable evidence gap rather than evidence for or against Level 3 architecture.

## 3 Sex-associated clinical disease trajectories

### 3.1 Survival and global disease severity

The female predominance of SSc is well established (3, 4), but disease burden is not distributed proportionally to prevalence. Men have more frequently exhibited diffuse cutaneous disease and selected severe internal-organ manifestations in several cohorts (5).

In a Toronto cohort of 959 patients, including 168 men, men more frequently had diffuse disease and ILD. Male sex remained associated with mortality after adjustment for age at diagnosis, disease subtype, ILD, anticentromere antibody status, and immunosuppressive treatment (5). Large multinational analyses have also identified male sex among variables associated with adverse outcomes (6, 7).

These associations are clinically important but should not be interpreted as evidence that male sex independently generates a more aggressive disease architecture. Observed sex associations may be influenced by differences in age at disease onset, autoantibody profile, cutaneous subtype, disease duration, environmental or occupational exposures, comorbidity, and treatment patterns (3–5). These variables should therefore be considered as potential confounders, mediators, or effect modifiers rather than assuming that an observed association with recorded sex represents a direct biological effect.

The appropriate conclusion is consequently narrower: sex provides prognostic information in several SSc populations, but the biological relationships responsible for that association remain incompletely defined.

### 3.2 Pulmonary fibrosis and pulmonary vascular disease

SSc-associated ILD provides an informative example of why cross-sectional sex differences and longitudinal disease behavior must be separated.

A *post-hoc* analysis of Scleroderma Lung Studies I and II identified sex-associated differences in pulmonary outcomes and biological profiles. Men in SLS II experienced less favorable changes in FVC and radiographic fibrosis and poorer long-term survival in selected analyses (17). An analysis integrating the focuSSced trial and SMART observational cohort likewise identified male sex as a prognostic marker of lung-function decline, while exploratory subgroup findings raised the possibility of sex-related treatment-effect heterogeneity with tocilizumab, without establishing a formal sex-by-treatment interaction (18).

However, a large EUSTAR analysis of 6,389 patients with SSc-ILD produced a more nuanced picture. Men had greater mortality but did not uniformly exhibit more rapid FVC deterioration (19). A subsequent pulmonary cohort of 393 patients likewise reported a higher prevalence and earlier diagnosis of ILD among men, whereas small-airway dysfunction was more prominent among women (20).

Consistent with this complexity, an exploratory serum proteomic study of 211 patients with SSc-ILD identified sex-associated differences among circulating proteins, but found no significant sex difference in time to FVC decline of >5% or >10%. Among 704 detected proteins, only autotaxin remained significantly different after correction for multiple testing (21). This provides a useful example of sex-associated molecular differences that do not necessarily translate into divergent longitudinal clinical trajectories.

SSc-associated pulmonary arterial hypertension provides another example. In the PHAROS registry of 131 patients with incident SSc-PAH, male sex was independently associated with mortality (22). By contrast, in a separate cohort of 378 patients with SSc-PAH, men had greater SSc disease burden and numerically poorer survival, but male sex was not an independent mortality predictor after adjustment (23).

Recent mechanistic work on SSc-associated pulmonary fibrosis has additionally highlighted sex-related differences in fibrotic biology and fibroblast responses, providing a biological rationale for further sex-stratified investigation (24). Such evidence remains complementary to, rather than a substitute for, prospective human interaction analyses.

These apparently divergent findings should not be forced into a single directional narrative. They instead demonstrate why SSc-MAF requires direct tests of sex-dependent relationships rather than assuming that sex-associated clinical severity necessarily reflects a distinct underlying biological architecture.

### 3.3 From cross-sectional phenotype to longitudinal trajectory

Most sex comparisons in SSc remain cross-sectional or outcome based. Yet identical measurements at a single time point can arise from different disease histories. A stable low FVC and a rapidly declining FVC may have very different biological implications despite similar values at assessment.

A trajectory-oriented analysis therefore requires repeated biological and clinical measurements. Relevant outcomes include FVC slope, quantitative HRCT progression, pulmonary vascular transition, skin-score trajectory, digital vascular complications, treatment exposure, and survival. The key SSc-MAF question is not simply whether the average trajectory differs by sex, but whether the relationship between biological states and subsequent trajectory differs.

For example, a fibroblast activation signature that predicts ILD progression equally in women and men would be prognostically useful but would not support sex-dependent architecture. Conversely, if the same baseline molecular state is coupled to different subsequent cellular transitions or clinical trajectories according to sex—and the interaction is reproducible after covariate adjustment—that would constitute coupling-level evidence.

### 3.4 What clinical evidence can—and cannot—establish

Clinical evidence therefore supports several distinct observations. SSc susceptibility is strongly female biased (3, 4), while the frequencies of selected clinical manifestations differ between women and men (5, 20). Male sex has also been associated with adverse outcomes in several longitudinal cohorts (5–7), although this relationship is not uniform across organ-specific populations (19, 23).

These clinical observations do not establish that molecular or multicellular SSc architecture differs by sex. They define the sex-associated phenotypic and longitudinal landscape that SSc-MAF seeks to explain, rather than proof of the proposed biological architecture (**Figure 2**).

**Figure 2:**
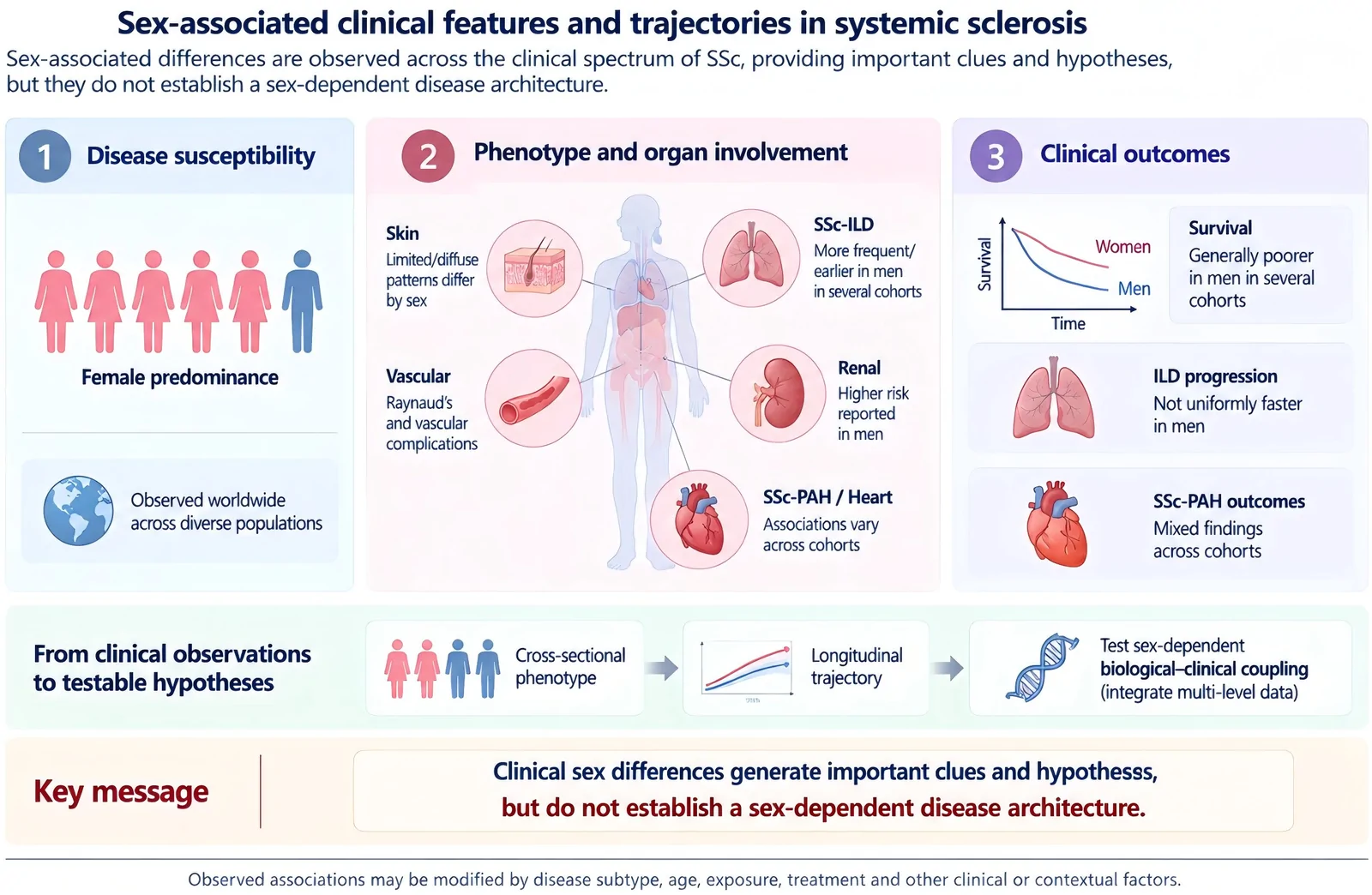
Sex-associated clinical features and trajectories in systemic sclerosis. Women are substantially more likely than men to develop systemic sclerosis (SSc), whereas sex-associated differences have also been reported in disease phenotype, organ involvement, and clinical outcomes. The magnitude, direction, and consistency of these associations vary across manifestations and cohorts. In several cohorts, men have shown a greater burden or earlier occurrence of SSc-associated interstitial lung disease (SSc-ILD), higher risk of selected severe organ manifestations, and poorer overall survival, whereas findings for ILD progression, SSc-associated pulmonary arterial hypertension (SSc-PAH), and other vascular outcomes are less uniform. These observations should therefore be interpreted as clinical signals that motivate longitudinal investigation rather than as evidence of sex-dependent biological architecture. Disease subtype, age, disease duration, treatment exposure, environmental factors, and other clinical or contextual variables may modify observed associations. Within SSc-MAF, the relevant next step is to determine whether longitudinal clinical trajectories are coupled to immune, vascular, stromal, or molecular states differently by sex, rather than simply whether individual clinical variables differ between women and men.

## 4 Biological disease architecture: evidence and hypotheses

### 4.1 From biological components to biological architecture

The distinction between a biological component and an architecture is central to SSc-MAF. A genetic locus, gene, pathway, cell population, metabolite, or epigenetic mark can differ between women and men without altering the relationships among disease processes.

A recent large sex-stratified GWAS illustrates this distinction particularly well. Analysis of 10,653 patients with SSc and 18,043 controls identified eight sex-associated susceptibility loci, including novel male- and female-associated signals (8). This represents strong direct evidence that the genetic determinants of SSc susceptibility can differ according to sex. However, even this study does not by itself demonstrate different multicellular or tissue-level SSc architectures.

We therefore define biological architecture as the organization of relationships among disease components. These relationships can be quantified as ligand–receptor communication, cellular neighborhood enrichment, network connectivity, covariance among molecular programs, temporal transition probabilities, or interaction terms linking biological states to clinical outcomes.

This definition prevents a common inferential error: accumulating multiple sex-associated findings and treating their coexistence as evidence of a sex-specific disease system. Architecture requires evidence of altered coupling, not merely multiple altered components.

### 4.2 Immune regulation: from X-linked signals to multicellular networks

The X chromosome provides one of the strongest biological rationales for investigating sex-associated immune regulation in SSc. Earlier studies identified abnormalities of X-chromosome inactivation among women with SSc (25).

More recently, Du and colleagues demonstrated altered X-chromosome inactivation of the TLR7/8 locus in SSc pDCs, accompanied by heterogeneity in pDC states and enrichment of type-I-interferon-associated populations (26). This study provides direct SSc evidence linking a sex-chromosome regulatory mechanism to an immune-cell phenotype. Studies outside SSc demonstrating biallelic TLR7 expression in subsets of female immune cells provide additional mechanistic context but should be regarded as indirect evidence (27).

Nevertheless, TLR7/8 XCI escape remains primarily component-level mechanistic evidence. It does not itself demonstrate a female-specific immune-network topology. Establishing the latter would require showing that altered X-linked signaling changes relationships among pDCs, B cells, T cells, monocytes, fibroblasts, or endothelial cells differently in women and men.

Interferon-related activation is well established in SSc (28, 29), while contemporary single-cell studies have revealed substantial immune and stromal heterogeneity linked to disease phenotype, stage, and autoantibody subgroup (10, 11). A systematic review of SSc single-cell studies illustrates the expanding cellular-resolution evidence base available for such analyses (30).

Recent SSc studies increasingly move beyond cellular composition toward direct multicellular relationships. CCL19^+^ fibroblast–CCR7^+^ T-cell crosstalk has been implicated in coordinated immunofibrotic signaling networks, with spatial and experimental evidence supporting the functional relevance of this axis (31).

Collectively, these observations identify plausible substrates for sex-dependent immune organization. They do not yet demonstrate that multicellular communication networks are systematically reorganized according to sex.

### 4.3 Fibrovascular remodeling: a candidate cross-compartment coupling

Vasculopathy and fibrosis are tightly intertwined in SSc (32). Endothelial dysfunction occurs early, while profibrotic mediators provide plausible links between vascular injury and fibroblast activation.

Single-cell studies have identified heterogeneous fibroblast populations and profibrotic states in SSc skin (9, 10). Systems-level analysis has further identified myofibroblasts and endothelial-to-mesenchymal-transitioning cells as communication hubs and implicated Hippo-pathway signaling in profibrotic mesenchymal differentiation (14).

Recent single-cell, spatial, and multimodal studies increasingly move beyond cellular composition toward direct characterization of fibrovascular and immune–stromal relationships. Spatial proteomics has identified heterogeneous vascular and perivascular populations and pathological vascular neighborhoods in SSc skin (12). ACKR1^+^ endothelial cells have been implicated in leukocyte recruitment, while infiltrating macrophages can promote activation of SFRP2/ASPN^+^ fibroblasts through profibrotic signaling (15).

Spatial multi-omic analysis has further demonstrated expansion of fibroblast–macrophage fibrotic niches and spatial dependency between these populations, with experimental CXCR4 inhibition attenuating fibrosis (13). Single-cell spatial phenotyping using cyclic *in-situ* hybridization has subsequently resolved additional fibroblast populations and pathogenic spatial niches in SSc skin (16). Multimodal analysis of early untreated dcSSc skin independently identified a proinflammatory vascular niche characterized by macrophage–fibroblast signaling and perivascular colocalization of fibroblasts, macrophages, and T cells (33).

Recent functional studies also reinforce the heterogeneity of profibrotic fibroblast states. RUNX1 expression has been localized to an SSc-enriched fibroblast subpopulation and associated with disease severity, while genetic and pharmacological inhibition reduced fibroblast activation and extracellular-matrix production (34).

These studies provide increasingly compelling evidence that multicellular, fibrovascular, and spatial coupling is a feature of SSc itself (12–16, 31, 33, 34). Critically, however, most were not designed to establish systematic female–male differences in those relationships.

SSc-MAF therefore treats fibrovascular coupling as a candidate architecture, while its sex dependence remains a testable hypothesis. The appropriate experiment is not merely to compare endothelial or fibroblast abundance between sexes. It is to determine whether endothelial–fibroblast, pericyte–endothelial, macrophage–fibroblast, or lymphocyte–stromal relationships differ reproducibly in strength, composition, location, or clinical relevance.

### 4.4 Metabolic and regulatory programs: an evidence gap rather than an established layer

Epigenetic dysregulation is well documented in SSc. Dermal fibroblasts show widespread DNA-methylation abnormalities (35), while genome-wide chromatin-accessibility studies demonstrate regulatory abnormalities in both endothelial cells and fibroblasts (36). More recent integrative studies linking fibroblast transcriptional states with epigenetic regulation further support the potential role of stable regulatory programs in SSc fibrosis (34).

These mechanisms provide plausible routes through which disease states could become stabilized over time. They also intersect conceptually with sex-chromosome dosage and hormonal signaling.

Metabolic reprogramming represents another potential stabilizing mechanism. Distinct metabolic programs can regulate macrophage polarization in inflammatory settings (37), providing a broader mechanistic rationale for investigating metabolic regulation of multicellular fibrotic states. However, direct evidence demonstrating reproducible sex-dependent metabolic coupling in human SSc remains sparse.

SSc-MAF therefore does not present metabolism as an established sex-dependent SSc architecture. Rather, metabolomic and epigenomic programs represent candidate regulatory layers whose contribution should be tested alongside cellular and spatial phenotypes.

### 4.5 Biological sex is not a single mechanistic variable

Recorded sex is an imperfect proxy for the biological mechanisms potentially relevant to SSc. Chromosomal complement, X-chromosome inactivation and escape, gonadal and peripheral hormone exposure, menopause, aging, hormone-receptor signaling, and tissue-specific regulatory programs may contribute differently across individuals and across the disease course (26, 27, 38).

Hormonal effects are particularly complex. A systematic review of sex hormones in SSc identified evidence consistent with both fibrogenic and vasodilatory actions of estrogens and substantial heterogeneity among available studies (38). Current evidence does not support reducing the female predominance of SSc to a single estrogen effect or using sex-hormone biology as a sufficient explanation for male–female differences.

Sex must also be distinguished from gender-related exposures and behaviors. Smoking, occupational exposure, healthcare access, diagnostic delay, adherence, socioeconomic factors, and treatment patterns can modify observed associations (4, 17). Autoantibody profile, ancestry, disease duration, age, menopausal status, cutaneous subtype, and therapy may additionally confound or mediate apparent sex effects (3–5, 38).

Future studies should therefore avoid interpreting a binary sex variable as a direct mechanistic measurement. Where feasible, sex should be analyzed alongside the biological and contextual variables that it may imperfectly represent. Together, these factors define the biological contexts through which sex may modify interactions among immune, vascular, stromal, and regulatory components, while remaining candidate mechanisms rather than established sex-dependent SSc architecture (**Figure 3**).

**Figure 3:**
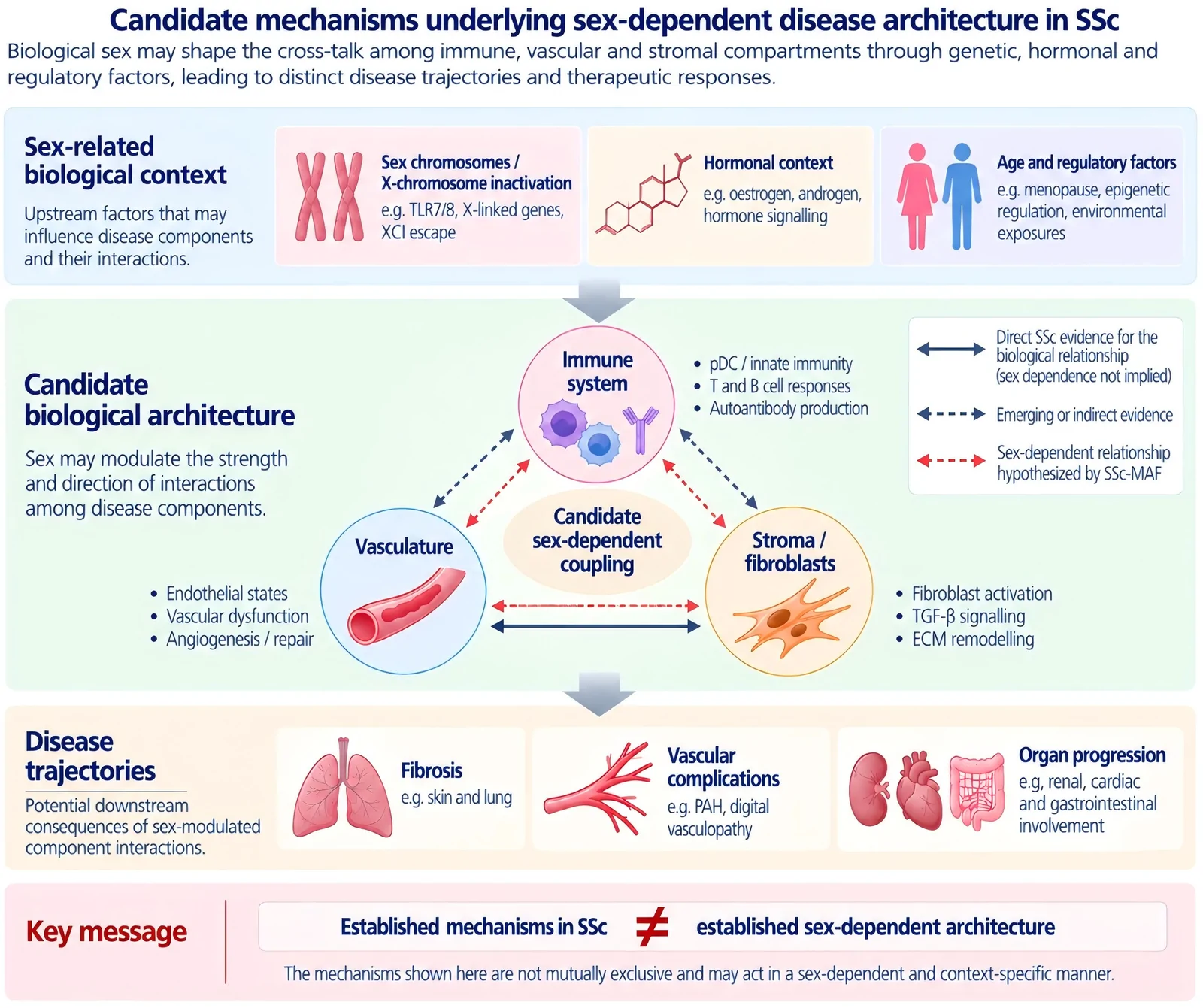
Candidate biological mechanisms underlying sex-dependent disease architecture in systemic sclerosis. Sex-related biological context—including sex chromosomes and X-chromosome inactivation (XCI), hormonal milieu, aging and menopausal status, regulatory programs, and environmental exposures—may influence immune, vascular, and stromal components of systemic sclerosis (SSc) and potentially modify the relationships among them. The central network highlights immune–vascular, immune–stromal, and vascular–stromal coupling as candidate relationships through which sex-associated biology could contribute to heterogeneous disease trajectories. Solid lines indicate biological relationships supported directly in SSc, without implying that their sex dependence has been established; dashed lines indicate emerging or indirect evidence; and red dotted lines denote sex-dependent relationships hypothesized by SSc-MAF and requiring empirical validation. X-linked immune mechanisms, including altered X-chromosome inactivation and TLR7/8-related signaling, provide examples of sex-linked component-level evidence but do not themselves establish sex-dependent disease architecture. Downstream fibrosis, vascular complications, and organ progression are shown as potential consequences rather than demonstrated products of sex-dependent coupling. The mechanisms depicted are not mutually exclusive, and their relative importance may vary according to disease subtype, stage, hormonal and aging context, treatment exposure, and other biological or environmental factors. Established mechanisms in SSc should not be interpreted as established sex-dependent disease architecture.

### 4.6 An evidence hierarchy for SSc-MAF

SSc-MAF uses the following hierarchy.

Level 1 — Sex-associated component: an individual clinical feature, genetic locus, gene, pathway, cell state, spatial abundance, metabolite, or regulatory mark differs according to sex.

Level 2 — Sex-dependent coupling: the relationship between at least two disease components differs according to sex, demonstrated by an interaction, network property, spatial relationship, trajectory transition, or equivalent measure.

Level 3 — Sex-dependent architecture: sex-dependent coupling is reproduced across independent cohorts, complementary biological layers, longitudinal observations, or perturbational experiments and provides information beyond additive sex effects and relevant covariates.

Most current sex-specific SSc evidence resides at Level 1. A growing body of SSc research demonstrates disease-level multicellular and spatial coupling without testing sex modification (12–16, 31, 33, 34). Direct evidence meeting the prespecified Level 2 criterion was not identified in the literature reviewed here, and Level 3 should currently be considered a hypothesis rather than an established feature of SSc. To make this distinction explicit, **Table 1** maps representative evidence across clinical, genetic, immune, fibrovascular, spatial, and regulatory domains according to the SSc-MAF evidence categories. Importantly, the table distinguishes direct sex-stratified SSc evidence from studies that establish disease-level coupling without testing its sex dependence, indirect mechanistic evidence, and framework-level hypotheses that remain to be tested.

## 5 Strategies for empirical validation of SSc-MAF

### 5.1 Study design principles: testing relationships rather than isolated differences

The central analytical requirement of SSc-MAF is explicit testing of interaction.

Demonstrating statistical significance in women but not in men does not establish that an association differs between women and men; the difference itself requires formal interaction testing (39, 40). Studies should therefore test sex-by-biological-state, sex-by-time, sex-by-treatment, or other relevant interaction terms directly and report effect sizes with uncertainty.

Sex-balanced recruitment is equally important. Because SSc is female predominant, conventional cohorts often contain too few men for reliable interaction testing. Purposeful enrichment of male participants may therefore be necessary for mechanistic studies even when such sampling does not reflect population prevalence.

Analyses should prespecify major confounders and avoid overfitting. Depending on the question, these may include age, disease duration, cutaneous subtype, autoantibody status, ancestry, smoking, menopausal status, treatment, baseline organ severity, and relevant comorbidities. High-dimensional analyses should additionally prespecify appropriate multiple-testing control, including false-discovery-rate procedures where applicable, and incorporate internal validation or cross-validation together with independent cohort replication whenever feasible.

### 5.2 Testing multicellular organization

Single-cell RNA sequencing, single-cell epigenomics, and high-dimensional cytometry can resolve cellular states obscured by bulk tissue measurements (9–11, 30).

Within SSc-MAF, however, cell abundance is only the first analytical level. A stronger test asks whether communication or state relationships differ according to sex. Candidate measures include ligand–receptor communication scores, sender–receiver centrality, pathway-specific information flow, cellular-state covariance, neighborhood connectivity, and state-transition probabilities.

Computational cell–cell communication estimates should be treated as hypotheses rather than direct measurements of physical signaling. Available inference methods differ substantially in databases, scoring functions, and assumptions (41). Stronger evidence would combine computational inference with spatial colocalization, receptor or protein validation, perturbation, or functional assays.

### 5.3 Testing spatial tissue organization

Spatial transcriptomics, spatial proteomics, imaging mass cytometry, multiplex immunofluorescence, and related technologies preserve tissue context and can identify cellular neighborhoods that cannot be reconstructed reliably from dissociated cells (12, 13, 16, 33).

A direct SSc-MAF test could compare women and men with respect to the frequency, composition, and spatial geometry of endothelial–pericyte, macrophage–fibroblast, lymphocyte–stromal, or other pathological neighborhoods. Importantly, demonstrating that a particular niche is enriched in SSc relative to healthy tissue would establish a disease-associated spatial architecture but not a sex-dependent one. The latter requires explicit sex-stratified or interaction-aware analysis.

### 5.4 Testing cross-layer coupling with multi-omic data

Multi-omic integration can test whether genomic, epigenomic, transcriptomic, proteomic, and metabolic states covary differently according to sex (42, 43). Latent-factor approaches such as MOFA^+^ provide one framework for identifying coordinated sources of variation across molecular layers (43).

SSc-MAF predicts that informative sex effects may occur in the relationships among layers rather than in their marginal distributions. For example, an inflammatory transcriptomic state might exhibit similar average abundance in women and men but show different coupling to fibroblast activation or vascular injury.

Replication is essential because high-dimensional integration is vulnerable to cohort-specific structure, batch effects, treatment imbalance, and overfitting.

### 5.5 Testing longitudinal biological–clinical coupling

Longitudinal cohorts provide the most direct bridge between biological architecture and disease trajectory.

Repeated biological sampling could be linked to FVC slope, quantitative HRCT, modified Rodnan skin score, pulmonary vascular progression, digital vascular events, treatment response, or survival. Mixed-effects models, joint models, trajectory analyses, and time-varying interaction models can test whether the relationship between a biological state and clinical evolution differs by sex.

A reproducible sex-by-biological-state interaction predicting subsequent outcome would represent stronger SSc-MAF evidence than a cross-sectional female–male difference in the same biomarker.

### 5.6 Experimental perturbation and causal testing

Observational coupling does not establish causality. Candidate mechanisms identified through human datasets should therefore be tested experimentally. Examples include perturbing an X-linked immune pathway and measuring downstream immune–stromal communication, manipulating endothelial or pericyte states and quantifying fibroblast responses, or altering a candidate regulatory program and testing whether network behavior changes differently under male- and female-associated biological contexts.

The increasing incorporation of perturbational validation into contemporary SSc studies—for example, CXCR4 inhibition in spatially identified fibroblast–macrophage niches (13), CCL19–CCR7 blockade in immunofibrotic coupling (31), and functional inhibition of fibroblast regulatory programs (34)—provides useful methodological precedents, although these experiments have not established sex-dependent effects.

Sex effects in experimental models should not automatically be extrapolated to human SSc. Their strongest role is to test whether a candidate relationship is biologically modifiable and to define mechanisms that can subsequently be examined in human tissues.

### 5.7 Computational models and artificial intelligence: prediction is not validation

Machine-learning approaches can integrate high-dimensional molecular, imaging, spatial, and clinical data (44). Their value within SSc-MAF lies in identifying reproducible patterns and generating testable predictions.

High predictive performance, however, does not demonstrate biological architecture. A model may exploit sex-correlated variables, technical artifacts, cohort composition, or treatment patterns without identifying a mechanistic relationship. Models intended to test SSc-MAF should therefore compare additive and interaction-aware formulations, use external validation, examine calibration and subgroup performance, and assess whether sex-dependent relationships provide incremental information beyond conventional covariates.

### 5.8 What would support—or fail to support—SSc-MAF?

Evidence would support an individual SSc-MAF proposition when a relationship among disease components differs reproducibly according to sex and survives appropriate adjustment, sensitivity analysis, and independent validation.

Support would become stronger if the same relationship were observed across complementary modalities or longitudinal data and stronger still if experimental perturbation altered the predicted downstream state.

Conversely, if adequately powered studies repeatedly demonstrate comparable relationships between women and men, that proposition would not be supported even when individual components differ by sex.

The framework should therefore be judged by its capacity to generate and survive empirical tests, not by the number of sex-associated observations that can be assembled. An integrated roadmap for this empirical validation strategy is summarized in **Figure 4**. To translate these principles into an operational and falsifiable framework, **Box 1** summarizes the five core propositions of SSc-MAF, together with their corresponding measurable endpoints and the findings that would support—or fail to support—each proposition. These criteria are intended to distinguish evidence for sex-dependent relationships among disease components from conventional observations of sex-associated differences in individual components.

**Figure 4:**
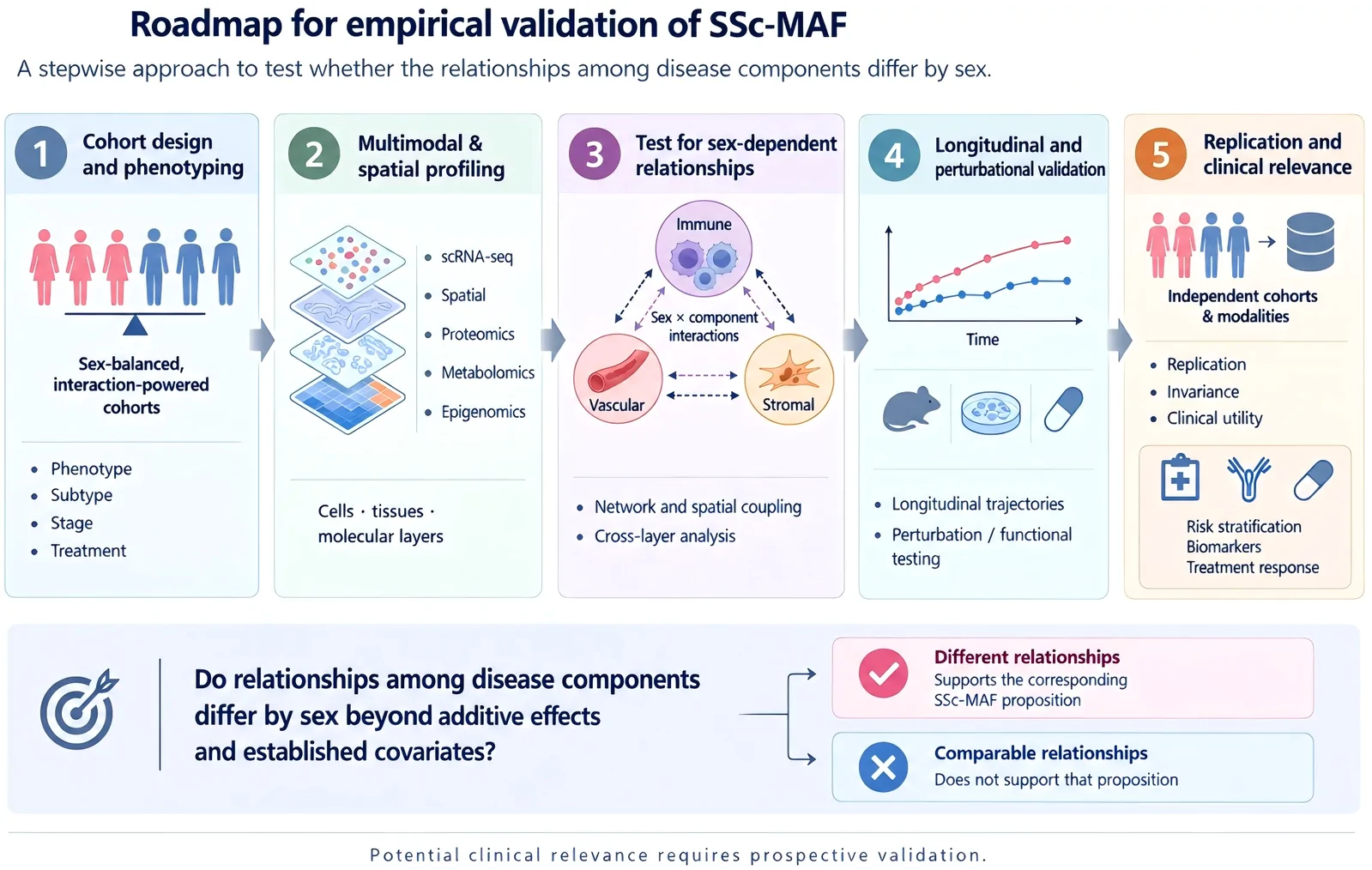
Roadmap for empirical validation of the Systemic Sclerosis–Multilayer Architecture Framework (SSc-MAF). Empirical evaluation of SSc-MAF requires study designs capable of distinguishing sex-dependent relationships among disease components from independent sex-associated differences. First, cohorts should include adequate representation of women and men and be sufficiently powered to test sex-by-component interactions, with careful phenotyping and consideration of disease subtype, stage, treatment exposure, and other relevant covariates. Second, multimodal and spatial profiling can characterize cellular, tissue, and molecular components and their cross-layer relationships. Third, candidate immune–vascular–stromal relationships should be formally tested using interaction-aware, network, spatial, and cross-layer analyses rather than inferred from differences in individual components. The network depicted in Step 3 represents a candidate structure to be tested, not an established sex-dependent architecture. Fourth, longitudinal analyses can assess temporal relationships, whereas experimental perturbation can provide functional support for selected candidate mechanisms; experimental perturbation should not be interpreted as evidence of clinical treatment efficacy. Finally, key findings should be evaluated in independent cohorts, tissues, platforms, or modalities, with appropriate separation of discovery and validation where feasible. Replication includes both reproducible sex-dependent relationships and reproducible invariance: the former would support the corresponding SSc-MAF proposition, whereas comparable relationships between women and men would not support that proposition. Any potential application to risk stratification, biomarkers, or treatment-response assessment would require prospective demonstration of clinical utility.

Box 1 **SSc-MAF: Testable propositions, measurable endpoints, and criteria for refutation**

**CENTRAL PREMISE** Sex-associated differences in individual disease components do not constitute evidence of sex-dependent disease architecture. Architectural evidence requires **reproducible sex-dependent relationships among disease components.**

**Table:** 

1	**Multicellular coupling** **Hypothesis** Communication among immune, vascular, and stromal cells differs according to sex. **Measure** Ligand–receptor interaction strength · network centrality · pathway information flow · experimentally validated sender–receiver effects **Supports** Reproducible sex-dependent interaction or network relationships after relevant adjustment. **Does not support** Differences restricted to cell abundance or gene expression without altered relationships.
2	**Fibrovascular and spatial coupling** **Hypothesis** Pathological cellular neighborhoods differ in composition or organization according to sex. **Measure** Neighborhood enrichment · intercellular distance · spatial co-occurrence · vascular–stromal adjacency · niche composition **Supports** Reproducible sex-dependent spatial relationships. **Does not support** SSc-versus-control spatial differences that are comparable between women and men.
3	**Cross-layer molecular coupling** **Hypothesis** Relationships among genomic, transcriptomic, epigenomic, proteomic, or metabolic programs differ according to sex. **Measure** Latent factors · cross-layer correlations · network modules · sex-by-factor interactions **Supports** Replicated sex-dependent cross-layer relationships. **Does not support** Isolated sex differences within one molecular layer.
4	**Longitudinal biological–clinical coupling** **Hypothesis** The relationship between biological state and subsequent disease trajectory differs according to sex. **Measure** Sex-by-biomarker-by-time interactions · trajectory-transition probabilities · biomarker–FVC/HRCT/vascular/skin/survival associations **Supports** Replicated interaction with prospective clinical evolution. **Does not support** Sex-associated outcome differences without corresponding differential biological coupling.
5	**Perturbational validation** **Hypothesis** Manipulating a candidate node alters downstream disease relationships in a context-dependent manner. **Measure** Perturbation-induced changes in signaling · cellular communication · spatial organization · matrix production · disease phenotype **Supports** Predicted downstream changes with mechanistic validation. **Does not support** Association without experimentally demonstrable influence on the proposed relationship.


**FRAMEWORK-LEVEL CRITERION**

Convergence across **independent cohorts** → **complementary modalities** → **longitudinal observations** → **experimental perturbation** progressively strengthens evidence for SSc-MAF. Conversely, consistently comparable intercellular, spatial, cross-layer, and biological–clinical relationships in adequately powered sex-balanced studies would weaken the central proposition that biological sex contributes to SSc disease architecture.

**Evidence assignment rule:**

Sex-associated component difference → **Level 1**

Demonstrated sex-dependent relationship between ≥2 components → **Level 2**

Reproducible sex-dependent coupling across independent cohorts, complementary modalities, longitudinal observations, or perturbational validation → **Level 3**

Failure to meet a higher-level criterion does not invalidate evidence assigned to a lower level.

## 6 Clinical and precision-medicine implications

### 6.1 From sex as a covariate to sex-aware risk assessment

Current evidence does not justify different SSc treatment algorithms solely on the basis of sex. Nevertheless, sex can remain clinically informative when interpreted alongside established prognostic variables.

Men are underrepresented numerically in SSc cohorts yet have greater frequencies of selected severe manifestations and adverse outcomes in several datasets (5–7, 17–20). Adequate male representation is therefore particularly important in prognostic studies and therapeutic trials.

The immediate translational implication of SSc-MAF is not sex-specific treatment but improved analytical design: sex should be examined as a potential effect modifier rather than included only as an additive adjustment variable.

### 6.2 Longitudinal biomarkers and risk stratification

SSc biomarker research increasingly aims to predict progression rather than classify established disease. SSc-MAF adds a specific question: does the relationship between a biomarker and progression remain stable across sex?

The SSc-ILD proteomic study illustrates the importance of this distinction: detectable sex-associated protein differences did not correspond to a significant sex difference in longitudinal FVC-decline endpoints (21).

A biomarker that performs similarly in women and men can be used broadly and would argue against sex-dependent coupling for that relationship. A biomarker whose association with progression differs reproducibly by sex would require mechanistic investigation and independent validation before clinical implementation.

The same principle applies to multimarker signatures, imaging biomarkers, molecular endotypes, and spatial tissue features.

### 6.3 Treatment response, pharmacology, and safety

Evidence for robust sex-by-treatment interactions in SSc remains limited. Exploratory analyses of SLS I/II and focuSSced/SMART raise the possibility of sex-associated differences in pulmonary trajectories or therapeutic response (17, 18), but subgroup findings should not be interpreted as established treatment-effect heterogeneity without appropriate interaction testing and replication (39, 40).

A prospective pharmacokinetic study of 34 patients with SSc, including only four men, found lower dose-normalized mycophenolic-acid exposure in men; this association was not reproduced for the unnormalized exposure measure (45). These findings are hypothesis-generating and do not support sex-specific dosing.

Future therapeutic studies should prospectively test sex-by-treatment interactions where biologically justified, while distinguishing pharmacokinetic exposure, efficacy, toxicity, adherence, and background disease severity.

### 6.4 Trial enrichment: from demographic stratification to biological enrichment

A more consequential use of SSc-MAF would be biological rather than demographic enrichment.

If future studies identify a reproducible sex-dependent multicellular or molecular state associated with progression, trials could enrich for the biological state itself rather than sex alone. This approach avoids assuming that all women or all men share a common mechanism.

Sex may then contribute to defining an endotype without becoming a surrogate for it.

### 6.5 What SSc-MAF could—and could not—change in clinical practice

SSc-MAF could improve research design by encouraging balanced recruitment, longitudinal sampling, interaction-aware analysis, spatial and multicellular validation, and explicit testing of whether biomarkers generalize across sex.

It could eventually contribute to risk stratification if sex-dependent relationships prove reproducible and clinically informative.

It cannot currently justify sex-specific surveillance intervals, drug selection, dosing, or therapeutic targets. Those applications require prospective clinical validation. Current management recommendations remain based primarily on organ involvement, disease phenotype, severity, progression risk, and treatment-specific evidence rather than sex-specific algorithms (46, 47).

The translational value of the framework therefore depends on whether it identifies relationships that improve explanation or prediction beyond established clinical factors—not on whether sex-associated differences can simply be catalogued.

## 7 Conclusion

Biological sex is consistently associated with important dimensions of SSc heterogeneity, including disease susceptibility, patterns of organ involvement, and clinical outcome (3–7). Recent large-scale genetic evidence further demonstrates sex-associated differences in selected susceptibility loci (8), while direct SSc studies identify sex-linked immune mechanisms such as altered X-chromosome regulation (26). Contemporary single-cell and spatial studies additionally demonstrate disease-level immune–stromal and fibrovascular coupling (12–16, 31, 33, 34), although their sex dependence remains largely untested.

Current evidence, however, is insufficient to conclude that women and men have distinctly organized molecular, multicellular, or tissue-level SSc architectures. Most available sex-specific evidence remains at the level of sex-associated components, and direct evidence meeting the prespecified criterion for sex-dependent coupling was not identified in the literature reviewed here.

This distinction defines both the value and the boundary of SSc-MAF. The framework does not presume that sex-dependent architecture exists; rather, it provides a structured path from component-level observations toward tests of sex-dependent coupling and, if replicated across complementary datasets or supported experimentally, toward evidence of sex-dependent disease architecture.

Equally important, SSc-MAF is intended to be falsifiable. If adequately powered and appropriately balanced studies demonstrate that intercellular relationships, spatial organization, cross-layer coupling, and longitudinal biological–clinical associations are comparable between women and men after accounting for relevant clinical and biological covariates, the central architectural proposition would not be supported, even if individual genes, cell states, or clinical phenotypes continue to differ by sex. Conversely, reproducible sex-dependent relationships that provide information beyond additive sex effects and established covariates would justify further mechanistic and translational investigation.

The current clinical implication is therefore one of research design rather than sex-specific management. Existing evidence does not justify different treatment or surveillance strategies solely on the basis of sex. The more immediate priority is to ensure adequate representation of both women and men, distinguish biological sex from its chromosomal, hormonal, aging-related, environmental, and gender-related correlates, and prospectively test sex-dependent relationships in longitudinal, single-cell, spatial, multi-omic, and experimental studies.

SSc-MAF should therefore be regarded as a testable framework rather than an established model of SSc pathogenesis. Its utility will ultimately depend not on whether additional sex differences can be identified, but on whether it reveals reproducible relationships that conventional component-level analyses fail to capture and whether those relationships improve biological explanation or clinically meaningful prediction. The framework should be retained, refined, or rejected according to that evidence.
